# Hepatitis B viral DNA integration occurs within three days of infection and is enhanced by ATR inhibition

**DOI:** 10.1016/j.tvr.2025.200332

**Published:** 2025-11-28

**Authors:** Dong Li, Jochen M. Wettengel, Harout Ajoyan, Vikki Ho, Gabriela Wu, Sarah Bae, Henrik Zhang, Delgerbat Boldbaatar, Jacob George, Mark W. Douglas, Thomas Tu

**Affiliations:** a Storr Liver Centre, The Westmead Institute for Medical Research, Westmead Hospital and The University of Sydney, Westmead, NSW, Australia; b Institute of Virology, Technische Universität München/Helmholtz Zentrum München, Munich, Germany; c German Center for Infection Research (DZIF), Munich Partner Site, Munich, Germany; d Sydney Infectious Diseases Institute, University of Sydney, Australia

**Keywords:** Hepatitis B virus, DNA repair pathways, Hepatocellular carcinoma, Insertional mutagenesis, Homologous recombination, Microhomology-mediated end joining, Non-homologous end joining, Double-stranded linear DNA

## Abstract

Chronic hepatitis B virus (HBV) infection is a major risk factor for hepatocellular carcinoma, with viral DNA integration into the host genome playing a pivotal role in oncogenesis. While HBV integration has been historically considered an event occurring late in a chronic infection, sensitive assays have detected integrations early infection. This study investigates the specific timing and molecular mechanisms of HBV DNA integration using a replication deficient HBV reporter system (HBV-Zeo) in HepG2-NTCP cells. Infection of this virus followed by positive selection led to cellular colony formation, showing that the input virus is the substrate that undergoes integration. By inducing DNA double-strand breaks via X-ray irradiation at specific timepoints after HBV infection, we observed a 2-3-fold increase in integration frequency when cells are irradiated between 16 and 76 h post-infection. Pharmacological inhibition of DNA repair pathways in this specific time window revealed that suppression of homologous recombination (HR) via ATR inhibitors significantly enhances integration rates (2.4–2.8-fold), while microhomology-mediated end joining (MMEJ) inhibition reduced integration to 17 % of untreated controls. These findings suggest that MMEJ plays a key role in HBV DNA integration occurring within hours of HBV infection. Together, our results advance understanding of HBV-associated hepatocarcinogenesis and may inform therapeutic strategies to disrupt viral integration and mitigate HBV-associated liver cancer risk.

## 1 Background

Chronic hepatitis B virus (HBV) infection affects ∼360 million worldwide [1], increases the risk of hepatocellular carcinoma (HCC, i.e. primary liver cancer) by ∼100-fold [2] and is the single most common driver of liver cancer (responsible for nearly 60 % of HCC cases). HCC is the third leading cause of cancer-related death worldwide, with approximately 830,000 deaths annually [3]. Due to the absence of specific symptoms during cancer development, HCC is often diagnosed at an advanced stage, resulting in poor therapeutic outcomes and a dismal 5-year survival rate of less than 20 % [4].

Integration of HBV DNA into the host cell is highly associated with liver cancers, as ∼90 % of HBV-associated liver cancers contain HBV DNA integrations, compared to 1 in ∼10^4^ cells in the non-tumour tissue [[5], [6], [7], [8], [9], [10], [11]]. Integration events have been reported to occur when breaks in the double-stranded DNA cellular genome are repaired through Microhomology-Mediated End Joining (MMEJ) and Non-Homologous End Joining (NHEJ) pathways, using HBV double-stranded linear DNA (dslDNA) as a substrate [[12], [13], [14]]. While NHEJ dominates as the primary double stranded break (DSB) repair pathway across all cell cycles, its error-prone nature facilitates HBV integration by introducing small indels at break sites. In contrast, MMEJ is considered a backup pathway and plays a critical role in rapid DSB repair during replication stress [15,16]. MMEJ involves using short tracts of homology to stabilise the ligation of two exposed DNA strands. Consistent with the use of MMEJ and NHEJ pathways, the cellular sites of HBV integrations are distributed widely across the entire human genome with short sequence homology between viral and host DNA in some integrations and indels evident in others [[17], [18], [19]].

Early studies of human HCC tissues using low sensitivity assays (e.g. Southern blot) suggested HBV integration occurs decades after initial infection [8,9,20]. This led to the prevailing model that integration is a late event, requiring long-term viral replication and cumulative genomic damage. However, emerging data challenge this paradigm. Studies using more sensitive PCR-based assays have detected HBV DNA integrations shortly after acute HBV infection [21,22] and in children with chronic hepatitis B [6]. In the woodchuck hepatitis virus (WHV) model, viral DNA integration has been reported 15 min after infection [21,23], although this is difficult to reconcile with human cell studies showing formation of cccDNA - the earliest marker of HBV entry into the nucleus - is not detectable until 12–16 h post-infection [24,25].

Although HBV DNA integration may occur within hours of infection, liver cancer may not occur until decades later. HBV DNA integration has been reported to drive liver cancer through two mechanisms: (1) *cis*-mediated effects (including insertional mutagenesis disrupting host gene function or regulation [[26], [27], [28]]; and (2) *trans*-mediated effects (e.g., persistent production of truncated and mutated viral proteins). However, the specific role of HBV DNA integrations in HBV-associated HCC (HBV-HCC) remains incompletely understood, as previous models do not recapitulate viral integration through authentic infection [[29], [30], [31]].

Here, we established an *in vitro* system to easily quantify and investigate factors associated with HBV DNA integration. Using a replication-competent reporter virus, we could assess the integrations resulting from first round HBV infection. We used this system to show that X-ray irradiation increases the rate of HBV DNA integration and precisely determined that integration occurs between 12- and 76-h post-infection. Moreover, we measured the effect of inhibitors of various DNA repair factors and found that blocking Ataxia Telangiectasia and Rad3-related protein (ATR) function (necessary for HR) induced a significant increase in integration rates.

## 2 Methods

### 2.1 Cell culture

A replication-deficient reporter HBV (HBV-Zeo) was generated by replacing the entire hepatitis B surface open reading frame (PreS1/PreS2/S) with a transthyretin promoter driven zeocin-resistance cassette. This HBV-Zeo construct maintains: 1) Core and HBx (but not polymerase and HBsAg) ORFs; 2) an intact ε packaging signal and direct repeats (DR1/DR2) for pgRNA packaging and 3) native HBV promoter/enhancer elements to preserve transcriptional regulation [32]. The zeocin marker enables selection of stable integrations in HepG2-NTCP cells. HBV-Zeo viral particles were produced and secreted by transfecting a helper cell line [33] trans-complementing polymerase and surface proteins through by the HBV-Zeo construct. Viral particles were purified by a streamlined heparin affinity chromatography and sucrose ultracentrifugation method as previously described [34].

HepG2-NTCP cells were infected with the reporter HBV-Zeo virus following a previously established protocol [35]. As a negative control, cells were treated with 100 μM Myrcludex B (Myr-B, an HBV entry inhibitor) from 15 min before infection to 18 h post-inoculation [36]. After inoculation, cells were washed three times with 1x DPBS (14,040,141, Gibco), and fresh DMEM media supplemented with 10 % v/v Fetal Bovine Serum (10099, Sigma-Aldrich), 20 mM L-glutamine (G7513, Sigma-Aldrich) and 2.5 % (v/v) DMSO (D2650, Sigma-Aldrich), and cells were incubated for 3 days. HepG2-NTCP cells were trypsinised using TrypLE Enzyme (12604021, ThermoFisher), re-seeded into 6-well plates and incubated for approximately 25 days to select HBV-infected cells in DMEM supplemented with 0.5 mg/mL Zeocin Selection Reagent (phleomycin D1; R25001, Invitrogen) to select HBV-infected cells. As cccDNA is cleared through mitosis [37], expanded colonies under these conditions therefore contain only cells with viral integrations (confirmed in our previous characterisation of these clones [38]).

To dissect the role of host DNA repair pathways in HBV integration, infected HepG2-NTCP cells were treated with inhibitors targeting distinct repair enzymes (Table 1). All compounds were prepared and applied according to manufacturer protocols. Concentration ranges were selected based on previously reported effective doses and cytotoxicity profiles [[39], [40], [41], [42]].

**Table 1:** The DNA repair pathways of compounds and concentrations used.

Compound	Molecule targeted	Pathway Targeted	Concentrations	Cat. No.
**ART558**	Polymerase θ (POLQ)	MMEJ	10, 20 or 40 nM	HY-141520, MedChemExpress
**AZD6738 (Ceralasertib)**	ATR	HR	0.5, 1 or 2 μM	HY-19323, MedChemExpress
**NU7441**	DNA-PKcs	NHEJ	0.25, 0.5 or 1 μM	HY-11006, MedChemExpress
**VE822 (Berzosertib)**	ATR	HR	25, 50, 100, 200 or 400 nM	HY-13902, MedChemExpress

ATR: Ataxia Telangiectasia and Rad3-related protein; DNA-PKcs (DNA-dependent protein kinase catalytic subunit; MMEJ: Microhomology-Mediated End Joining; HR: Homologous Recombination; NHEJ: Non-Homologous End Joining.

Inhibitors were applied at two critical timepoints: 1) Pre-integration treatment: cells were pretreated with inhibitors for 24 h prior to HBV-Zeo infection, and at 72 h post-infection (hpi); 2) Co-integration treatment: inhibitors were added at 4 hpi and maintained until 72 hpi to target the window when integration occurs.

### 2.2 Quantification of HBV DNA copy number per cell

Total cellular DNA was extracted from cell pellets using the QIAGEN DNeasy Blood and Tissue Kit (69506, Germany) according to the manufacturer's instructions and then eluted in 20 μL of elution buffer. DNA concentration was quantified using NanoDrop™ 2000 spectrophotometer and stored at −30 °C for further experiments.

Digital droplet PCR (ddPCR) was used to quantify the copy number of HBV DNA integrations. 500 ng of total DNA was first digested with *Eco*RI-HF (R3101S, New England Biolabs) in a 20 μL reaction containing 1x Cutsmart Buffer and 10 units *Eco*RI-HF. 25 ng of digested DNA was added to a 20 μL of ddPCR mastermix consisting of 1X ddPCR Multiplex Supermix for Probes (12005910, Bio-rad), and target-specific primers and probes (sequences and final concentration listed in Table 2). The samples underwent droplet generation using the Bio-rad QX200™ Droplet Generator Cartridges (1864008, Bio-rad), Oil (1863004, Bio-rad) and QX200™ Droplet Generator. 40 μL of oil-emulsion droplets were transferred into a ddPCR plate and amplified by PCR (initial denaturation at 95 °C for 10 min, followed by 40 cycles of a denaturation at 95 °C for 10 s, an annealing at 54 °C for 15 s and an extension at 68 °C for 20 s, deactivation at 95 °C for 10 min). Droplets were analysed by a Bio-rad QX200™ Droplet Reader and resultant data was analysed in Quantasoft. The concentration of HBV DNA was normalized against RNase P, known to be present as two copies per cell [43].

**Table 2:** The sequence of primers and probes targeting Zeocin resistance and HBx gene.

Primer Gene Target	Primer Sequence	Final Concentration
Zeo-Resistance (Zeo-R) targets zeocin resistance gene in integration model	F: 5′- CAAGTTGACCAGTGCCGTTC-3′	225 nM
	R: 5′- CTCGCCGATCTCGGTCATG-3′	
	Probe:/56-FAM/GGACACGACCTCCGACCAC/3BHQ_1/	
RNaseP (VIC-labelled)	TaqMan™ copy Number Reference Assay, human, RNaseP (4403326, ThermoFisher)	1X

### 2.3 X-ray irradiation

X-ray irradiation was performed with a cabinet X-ray irradiation system X-RAD320 Biological Irradiator (Precision X-Ray Inc., North Branford, CT) at the Westmead Institute for Medical Research. X-ray was generated with an operating voltage of 320 kVp and a tube current of 12.5 mA, using a 2.0 mm Al filter, at a dose rate of approximately 1.15 Gy/min. Cells were exposed to X-ray irradiation at doses of 0.08, 0.4, 2 or 4 Gy, as described previously [44].

### 2.4 Quantifying HBV infection rate

Total HBV DNA or HBeAg secretion were used to quantify HBV infection. To quantify total HBV DNA DNA from HBV-Zeo infected HepG2-NTCP cells, digital PCR (dPCR) was performed using the QIAcuity One platform (Qiagen). Total DNA was extracted and digested as above, and 50 ng of digested DNA was added to 40 μL of dPCR mastermix reaction containing 1X QIAcuity Probe PCR Master Mix (250015, QIAGEN) and primers specific for HBx and RNaseP (sequences and final concentration listed in Table 2). Reactions were loaded into QIAcuity Nanoplates (26k, 24-well format, 250001) and sealed. PCR amplification was carried out on the QIAcuity One instrument (5plex) using the following thermal cycling conditions: Initial denaturation at 95 °C for 2 min, followed by 40 cycles of denaturation at 95 °C for 10 s, annealing at 54 °C for 15 s, extension at 68 °C for 20 s, and deactivation at 95 °C for 10 min. After amplification, the partitioned reactions were imaged and analysed using the QIAcuity Software Suite (v3.1). HBV DNA copy numbers per cell were calculated using Poisson distribution-based analysis provided by the QIAcuity software (v3.1).

Secreted Hepatitis E Antigen (HBeAg) from cells was measured using the HBeAg ELISA Assay kit (WB-2496, Wantai) according to the manufacturer's instructions. Standards were generated using serum from an HBV-positive patient (Genotype B, immune-tolerant, HBeAg-positive, NA treated, HBeAg measured against WHO standard = 1888 ng/μL). Absorbance measurements (Optical Density, OD) of the samples were obtained using the SpectraMax iD5 Multi-Mode Microplate Reader (Molecular Devices) at two wavelengths: 450 nm to quantify HBeAg and 630 nm to measure background absorbance. Background absorbance was subtracted to normalise [OD (450 nm)- OD (630 nm)] and the blank-corrected values used to determine the lower limit of detection (LLOD). Absolute HBeAg concentration was calculated based on the standard curve. For samples exceeding the linear range, a 1:5 initial dilution in assay buffer was performed, followed by iterative dilutions (1:2 to 1:10) until OD values fell within the standard curve's linear range (20–1000 ng/μL). Final concentrations were adjusted for dilution factors.

## 3 Results

### 3.1 Establishment of a system to quantify *de novo* HBV DNA integration rates

**Fig. 1:**
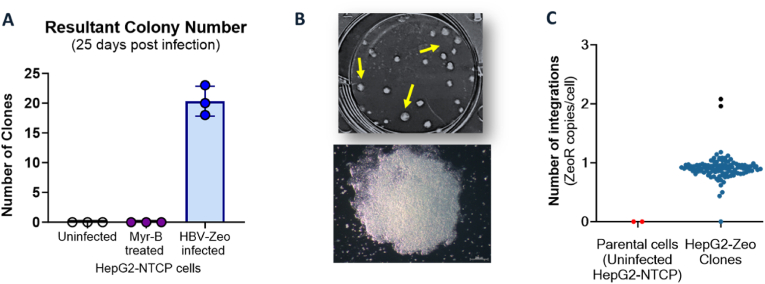
**Generation of HepG2-NTCP cell clones harbouring *de novo* HBV DNA integrations.** (A) HepG2-NTCP cells were infected with HBV-Zeo (with or without the presence of100 μM Myrcludex B), and then selected with Zeocin (250 μg/mL) to isolate clones. (n = 3 biological replicates). **(B) Phase microscopy of resultant clones.** (Top) Yellow arrows indicate the representative colonies (23 colonies 6-well plate shown). (Bottom) Microscopy image of one colony at 4× magnification. Scale bar: 200 μm. **(C) Quantification of integrations in HepG2-NTCP HBV-Zeo Clones.** Integrated HBV DNA was quantified by ddPCR, with primers specific for the Zeocin resistance gene (Zeo-R) with reference gene RNase P (2 copies/cell) for normalisation. Red points indicate uninfected parental cells (negative control, n = 2), and blue points represent HepG2-NTCP Zeo clones (n = 106). The absolute copy number was determined by the nearest whole number within the Poisson error (1 copy = blue dot, 2 copy = black dot).

HepG2-NTCP cells were infected with HBV-Zeo and selected with Zeocin to enrich cell colonies harbouring *de novo* HBV DNA integrations as previously described [38] (Fig. 1A). As a negative control, cells were pre-treated for 15 min with Myrcludex B (Myr-B) (100 mM), an HBV entry inhibitor [36]. Myrcludex B was also included in the infection media for 18–20 h during inoculation. No colonies were observed in Myr-B-treated controls, indicating that HBV entry is essential for integration and colony formation (Fig. 1B).

Assuming a starting population of 5 × 10^5^ HepG2-NTCP cells and ∼30 % infection, we estimated ∼1.5 × 10^5^ infected cells were present in each well [35,45]. Each well generated on average 20 integration-containing clones, corresponding to an observed integration frequency of ∼1 in 7500 infected cells, consistent with previously reported integration rates of ∼1 per 10,000 infected cells [11,12,46].

Colonies from multiple infections were isolated (106 single cell-derived clones, in total). ddPCR analysis showed that each clone contained 1 or 2 integrations per cell with no HBV DNA detected in parental cell controls (Fig. 1C), normalized to the diploid reference gene RNase P, is consistent with a pattern of genomic integration. This and our previous data characterizing clones developed using this method suggest there is limited possibility that these clones contain significant levels of cccDNA after selection [38]. We have previously shown that the level of HBV DNA detected by direct PCR is the same level as measured by either ddinvPCR or invPCR (integration-specific PCR assays), showing they are not derived from cccDNA. Targeted HBV DNA sequencing of these clones was able to find the exact virus-cell junction in all clones, directly showing that these sequences are integration derived. Thus, the quantified colonies are very likely the result of stable genomic integration events.

### 3.2 HBV DNA integration occurs between 16 and 76 hpi post-infection

We used X-ray irradiation to rapidly induce double stranded DNA breaks, which have been reported to occur within 20 s of irradiation [47] and peak ∼30 min post-irradiation [48]. We found that a dose of 0.4 Gy was able to induce DNA breaks in HepG2-NTCP cells while not affecting downstream cell mitosis (**Supplemental materials**).

**Fig. 2:**
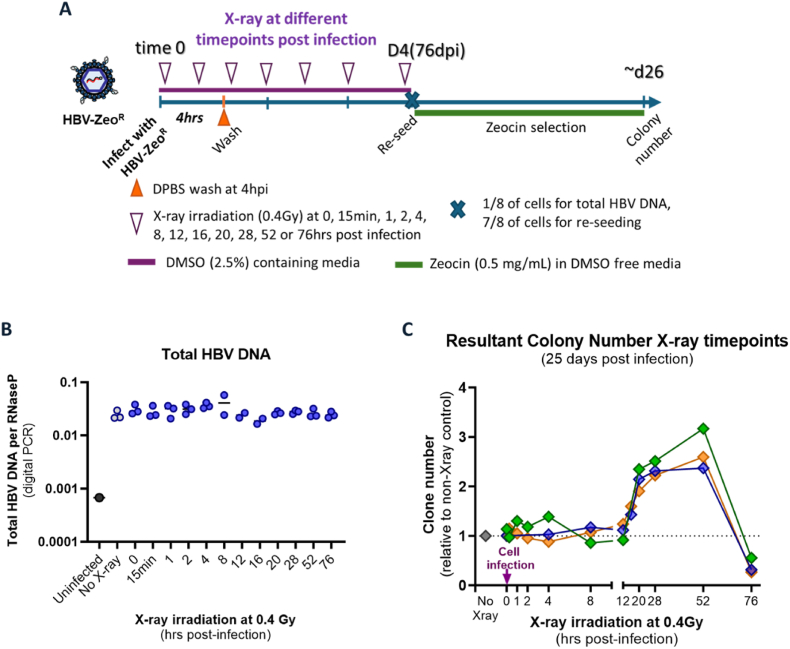
**X-ray irradiation increases HBV DNA integration between 16 and 52hpi. (A) Experimental outline.** HepG2-NTCP cells were irradiated at the indicated time points (0–76 hpi) following HBV-Zeo infection, and Zeocin-resistant clones were quantified after selection**. (B) Total intracellular HBV DNA levels** were measured by dPCR of total DNA extracts at 4 days post infection after X-ray irradiation at each timepoint. Each data point represents the mean of three replicates. (**C) Integration rate** was measured by the number of clones resultant after zeocin selection. Each data point represents the mean of three replicates, with results from three independent experiments plotted in green, blue, and orange, respectively. A dashed horizontal line at Y = 1 indicates the baseline level of non-irradiated controls.

As cellular double stranded DNA breaks are the substrates for HBV DNA integration [12], we posited that increasing the number of DNA breaks by X-ray irradiation would increase the number of integration events when these two events coincide. Thus, to define the exact timepoint when HBV DNA integration occurs post-infection, HepG2-NTCP cells were infected with HBV-Zeo for a short duration (4 h) to increase temporal resolution (Fig. 2A for experimental outline) and then irradiated with X-rays at various time points after infection: 0, 15 min, 1, 2, 4, 8, 12, 16, 20, 28, 52, or 76 h post-infection (hpi). Colony formation was used as a measure of HBV DNA integration frequency.

The rate of HBV infection appeared unaffected by X-ray irradation: intracellular total HBV DNA levels (quantified by dPCR) were not significantly different between cells irradiated at different time points (Fig. 2B). Thus, time points could be directly compared with one another without needing to consider differences in infection rate.

Cells irradiated at times between 0 and 12 hpi did not show significantly different rates of colony formation compared to non-irradiated controls. (Fig. 2C). A small increase in colony formation rate was observed in cells irradiated at 16 hpi, increasing between 28 and 52 hpi (showing a 2-3-fold increase compared to non-irradiated controls). A sharp decline was observed in cells irradiated at 76 hpi (∼30–50 % of the non-irradiated cells). Thus, we find that HBV DNA integration occurs in a specified window of 12–76 hpi.

### 3.3 HBV DNA integration increases with inhibition of ATR

**Fig. 3:**
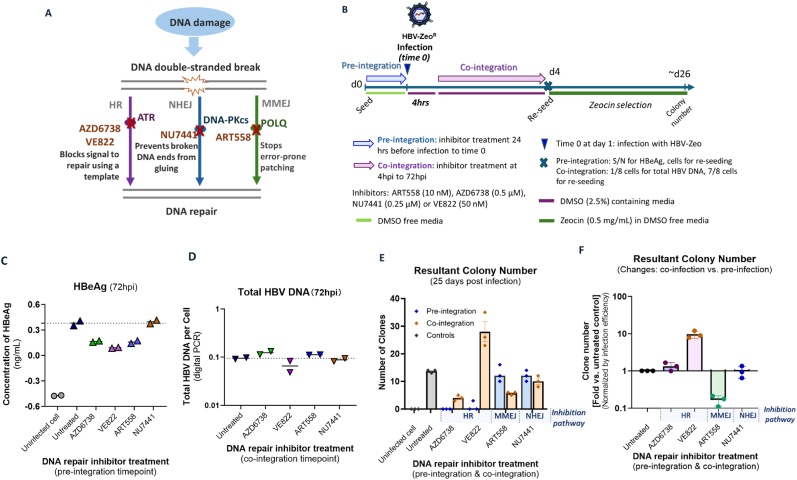
**DNA repair pathway inhibition and its impact on HBV integration. (A) Schematic representation of the three principal double-strand break repair pathways** - homologous HR, NHEJ, and MMEJ. Key regulatory proteins (ATR, DNA-PKcs, and POLQ) are shown with red “X” symbols indicating sites of pharmacologic inhibition. **(B) Experimental workflow outlining two treatment phases: a pre-integration window** (−24 to 0 hpi), where inhibitors may alter baseline cellular repair capacity before viral entry, and **a co-integration window** (4–72 hpi), where inhibitors act during the known period of HBV DNA integration. **(C). Assessment of viral infection at pre-integration treatment time-points** was measured by dPCR of total intracellular HBV DNA, while **(D) viral infection at co-integration treatment time-points** was measured by HBeAg secretion**. (E) Absolute integration rate** in untreated controls (black), pre-integration treatment (blue) and co-integration treatment (orange) was measured by enumeration of resultant clones after selection**. (F) Integration rates were normalized** against pre-integration treatment controls, to account for inhibition of infection due to inhibitors and any cell death. All graphs show mean ± SEM from 2 to 3 independent biological replicates, each plotted as an individual data point.

Pharmacological inhibition of DNA repair pathways may modulate HBV DNA integration by either prolonging the persistence of DNA DSBs or redirecting repair toward error-prone mechanisms during the integration window. To evaluate which pathways may be involved in the HBV DNA integration process, we employed small-molecule inhibitors targeting distinct repair enzymes (Fig. 3A): ART558 (10 nM) inhibiting POLQ, a key molecule in MMEJ promoting error-prone repair of DSBs; AZD6738 (0.5 μM) and VE822 (50 nM) inhibiting ATR, a key molecule in HR that regulates DNA damage signalling; and NU7441 (0.25 μM) inhibiting DNA-PKcs, a core component of classical NHEJ. These concentrations were selected based on cytotoxicity assays, ensuring minimal impact on cell viability while effectively inhibiting their respective pathways (**Supplemental materials**). It is important to note that when a particular DNA repair pathway is inhibited, the other two may increase to repair the increased number of DNA breaks [16,49,50].

To dissect the role of DNA repair pathways in integration, infection of HepG2-NTCP cells with HBV-Zeo was repeated and inhibitors was applied at two critical timepoints (Fig. 3B).

1) Pre-integration (24 h before infection to time 0 - defined as the time of inoculation): this accounted for any changes due to reductions in cell viability or ability to undergo mitosis due to application of the drugs (assessing baseline effects on cell fitness and viral entry).
2) Co-integration (4–72 hpi): this measured the effect of inhibitors directly at the peak times of integration (targeted the window in which integration occurs to evaluate direct impacts on HBV DNA integration).

We first determined effects of the inhibitor treatment on HBV infection rate. For the pre-integration treatment, we quantified HBeAg secretion as a functional measure of successful cccDNA establishment and transcriptional activity. For the co-integration treatment, we measured total intracellular HBV DNA levels to directly quantify the pool of viral DNA substrates available for integration, thereby avoiding potential confounders from inhibitor effects on viral gene expression. In both treatment windows, we observed only minor changes (<2-fold) in infection establishment compared to untreated controls (Fig. 3C and D). Consequently, we normalized subsequent colony formation counts to HBeAg levels (pre-integration) or HBV DNA levels (co-integration) to account for these slight variations in infection efficiency.

When comparing untreated cells to cells treated with DNA repair inhibitors at pre-integration timepoints, we found that inhibition of the ATR significantly reduced colony formation compared to untreated controls (mean clone number AZD6738: 0; VE822: 1.0 vs untreated cell: 13.7) (Fig. 3E). This suggests that ATR inhibition alters viral entry pathways or conversion of dslDNA forms into cccDNA, consistent with previous studies [51,52].

We found evidence that MMEJ was a likely pathway for HBV DNA integration, when comparing the change between pre-integration treatment and co-integration treatment. ATR blockade significantly increased integration frequency (mean difference VE822: 27, p < 0.001, paired *t*-test, standardized effect size = 10.7; AZD6738: 5.4, p < 0.001, paired *t*-test, standardized effect size = 24.5). In contrast, MMEJ/NHEJ inhibition showed either neutral (NU7441: 2, p = 0.41) or suppressive effects (ART558: 6.4, p = 0.03). When represented as a ratio between co-integration treatment and pre-integration treatment (Fig. 3F), integration rates increased with ATR inhibition via VE822 (mean: 9.62 ± 2.13-fold, p < 0.0001) or AZD6738 (1.33 ± 0.34- fold, p = 0.27). Conversely, inhibition of POLQ (a component of MMEJ) using ART558 reduced integration to 17 % of untreated controls (0.17 ± 0.05-fold, p = 0.0002), suggesting that MMEJ is a key pathway for HBV DNA integration.

Inhibition of DNA-PKc (a component of the NHEJ pathway) using NU7441 had no observable effect on integration rates (0.97-fold, p = 0.99), indicating either minimal involvement of NHEJ or redundancy in the pathway (one-way ANOVA, R squared = 0.94).

## 4 Discussion

Our study provides critical insights into the molecular drivers of HBV DNA integration, addressing the hypothesis that initial integration occurs early during infection and is mediated by specific host DNA repair pathways. Using a replication-deficient HBV reporter system, we demonstrate that HBV DNA integration mainly occurs within a defined window of 12–76 hpi, independent of intracellular viral replication in this system. While the use of X-ray irradiation to induce DSBs could potentially influence the absolute frequency of integration, the clear temporal profile of increased colony formation (occurring between 16 and 52 hpi), specifically identifies the window during which the input viral DNA is competent to serve as a substrate for DSB repair. This timeline is further corroborated by the finding that inhibition of the MMEJ pathway is most effective at suppressing integration when applied during this same window (4–72 hpi, Fig. 3F). Together, this data shows that integration is coincident with the formation of cccDNA (12–72 hpi) [25]. This supports the conclusion that integration originates from input viral genomes rather than from second-round replicative intermediates, consistent with prior studies using HBc-deficient HBV that is unable to undergo productive replication [24]. We observed no evidence of integration occurring within 15 min of infection, as previously reported in the woodchuck model [21,23,53], potentially due to species-specific differences.

The apparent lack of integration beyond 72 hpi in our system should not be interpreted as an absolute limit for integration events *in vivo*. It is important to note that our reporter virus is replication-defective, restricting our results to assessing a single round of infection; once the input viral genomes are processed, no new viral DNA substrates for integration are generated. In contrast, chronic HBV infection in patients is likely characterized by continuous cycles of hepatocyte infection, which would provide ongoing chances of HBV DNA integration. Thus, while our data demonstrate that integration occurs early after initial entry, the ongoing hepatocellular infection *in vivo* is likely to cumulatively drive integration events over time and contribute to the high integration burden observed in patient livers.

Exactly how much integration occurs over the course of a chronic infection is difficult to calculate, even with this new data. For example, there is little known to what extent super-infection exclusion occurs with the chronically infected liver [54]. For human HBV, this could range from complete to non-existent. Given the strong dependence of new infection events on the integration rate, this single fact makes it very difficult to even theoretically calculate the ongoing rate of integration. Integration rates may also depend on the level of inflammation (driving clonal expansion of cells with integrations), the extent to which cells with integrations are recognised and cleared by the immune system, whether integration rates linearly increase with viral load, etc. Our results provide a single figure in this highly-complex system which may be used in future to calculate the level of integration rate over the course of a chronic or acute HBV infection.

We showed that pharmacological inhibition of ATR with AZD6738 and VE822 significantly increases integration rates. Pharmacological inhibition experiments revealed two key mechanistic findings: (a) ATR inhibition significantly increased integration rates when applied at pre-integration timepoints, consistent with ATR promoting homologous recombination and thereby limiting error-prone repair, and (b) POLQ inhibition strongly suppressed integration, suggesting that MMEJ is a likely pathway involved in HBV DNA integration. Indeed, this is consistent with previous sequencing data showing that HBV DNA integrations have a higher than expected chance to show seqeunce microhomology at the virus-cell DNA junction [[17], [18], [19]]. In addition, the increased integration rate under ATR inhibition could reflect an elevated abundance of nuclear viral DNA free ends, as ATR inhibition has been linked to increased deproteinized HBV rcDNA forms [52]. Whether these forms actively participate in integration remains unclear, future analysis of viral integration ends under such conditions may resolve this question.

Collectively, our findings suggest that MMEJ is a likely pathway for HBV DNA integration and that it occurs very early in an infection. These insights not only advance our understanding of HBV persistence but also reveal actionable therapeutic targets. First, these results underscore the importance of early intervention (e.g. vaccination and antiviral treatment) to prevent ongoing infection events and therefore reducing integration rates. Since integration begins almost immediately after infection and likely accumulates over time, early suppression of viral replication with NA could significantly reduce the long-term integration burden and cancer risk. Indeed, this supports recent calls for expansion of NA treatment for both health benefits and limiting anxiety over disease progression [55,56], even in apparently healthy younger people (not currently recommended in many clinical management guidelines).

Second, our identification of MMEJ a likely pathway for HBV DNA integration reveals novel therapeutic strategies. The development of anti-integration agents, such as POLQ inhibitors or other MMEJ antagonists, holds promise. These would likely be used in combination with existing antivirals to target both new integration events and ongoing viral replication. Furthermore, our replication-deficient reporter system provides an ideal platform for drug discovery pipelines to screen for selective anti-integration compounds.

Third, the landscape of HBV therapy is evolving. Novel epigenetic therapeutics designed to silence both cccDNA and integrated HBV DNA have entered clinical trials. If effective, these agents could target the transcriptional activity of established integrations, addressing a key driver of oncogenesis and potentially contributing to a functional cure.

Several limitations of our experimental system should be acknowledged. Our model depends on the zeocin resistance gene to be included in the integration. Long-read sequencing studies of human liver tissues from HBV patients have shown that the majority of integrations include HBsAg ORF (which is replaced by the zeocin resistance gene in our system), there is a subset of integrations (∼20 %) that do not [57]. Whether these represent integrations occurring through a different mechanism is unknown, but cannot be studied using our platform.

Secondly, our reporter HBV lacks HBsAg expression. In natural infections, integrated HBV DNA often drives constitutive HBsAg production, which may induce endoplasmic reticulum stress and promote hepatocyte apoptosis [58,59]. However, we have shown in previous work that the expression of HBsAg is relatively low during the time period in which integration is occurring and is only detetable by highly sensitive RNAscope assays 72hr post-infection. Thus, we suggest that the HBsAg expression would not have a strong contribution on integration rates in this system.

The suggestion that MMEJ is the dominant pathway is based on pharmacological evidence and sequencing results from previous studies. While the significant suppression of integration upon POLQ inhibition and its potential enhancement upon ATR blockade provide robust functional evidence, this study lacks direct mechanistic evidence. Knocking out MMEJ pathways in future studies (e.g. using CRISPR approaches) may allow confirmation of our hypotheses.

Looking forward, our findings provide a framework for several research directions: (1) investigating extrinsic triggers of integration (e.g., inflammation or genotoxic stress) to inform adjunctive therapies that lower integration risk; and (2) developing therapeutic strategies aimed at mitigating HBV-driven genomic instability and ultimately preventing HBV-associated hepatocarcinogenesis. Future work could translating these insights into strategies to mitigate HBV-driven genomic instability and liver cancer.

## CRediT authorship contribution statement

**Dong Li:** Writing – review & editing, Writing – original draft, Visualization, Validation, Investigation, Formal analysis. **Jochen M. Wettengel:** Writing – review & editing, Methodology, Investigation. **Harout Ajoyan:** Writing – review & editing, Software, Methodology, Investigation, Formal analysis, Data curation. **Vikki Ho:** Writing – review & editing, Methodology, Investigation. **Gabriela Wu:** Writing – review & editing, Methodology, Investigation. **Sarah Bae:** Writing – review & editing, Methodology, Investigation. **Henrik Zhang:** Writing – review & editing, Methodology, Investigation. **Delgerbat Boldbaatar:** Writing – review & editing, Methodology, Investigation. **Jacob George:** Writing – review & editing, Supervision, Resources, Funding acquisition, Ulrike Protzer, Writing – review & editing, Supervision, Resources, Funding acquisition. **Mark W. Douglas:** Writing – review & editing, Supervision, Resources, Funding acquisition. **Thomas Tu:** Writing – review & editing, Writing – original draft, Supervision, Project administration, Methodology, Investigation, Funding acquisition, Data curation, Conceptualization.

## Funding

This work received funding from the following sources: the Paul and Valeria Ainsworth Precision Medicine Fellowship (TT); GESA 2025 Lawrie Powell AC Mid-Career Grant (TT); the Australian National Health and Medical Research Council Ideas, Program and Investigator Grants APP2002565 (MD, TT, SB), APP20021586 (MD, TT, SB), APP1053206 (JG), APP2001692 (JG), APP1107178 (JG), APP1108422 (JG), and APP1196492 (JG); Australian Centre for HIV and Hepatitis Virology Research (TT, MD); Cancer Institute, NSW grant 2021/ATRG2028 (JG); and the Robert W. Storr Bequest to the Sydney Medical Foundation (JG). Bioinformatics analysis (BG) was performed at the Westmead Scientific Platforms, which are supported by the Westmead Research Hub, Cancer Institute New South Wales, the National Health and Medical Research Council, and the Ian Potter Foundation.

## Declaration of competing interest

Thomas Tu serves as consultant to Excision BioTherapeutics and Kerna Ventures with fees to his institution, as paid consultant to Gilead Sciences and GSK, and received payment for speaking engagements from GSK, ASHM, and the Singapore HBV Cure conference. All other authors declare no relevant conflicts of interest.

## Data Availability

Data will be made available on request.
