# Supplementary material for: Hepatitis B viral DNA integration occurs within three days of infection and is enhanced by ATR inhibition
Source: Tumour Virus Res. 2025 Nov 28;20:200332. doi: 10.1016/j.tvr.2025.200332 (PMC12719184; doi:10.1016/j.tvr.2025.200332)
Supplement: Multimedia component 1 [file mmc1.docx]

**Supplemental Materials**

**Methods**

**Cell viability** **analysis**

Cell viability was assessed using DAPI viability staining and flow cytometry. At the indicated time points, cells were harvested by trypsinization and pelleted at 300 × g for 5 minutes. The cell pellet was washed once and resuspended in wash buffer (DPBS supplemented with 2% v/v Fetal Bovine Serum). Approximately 90 µL of the cell suspension was incubated with 10 µL of diluted DAPI solution (final concentration 1:10 of stock) (Beckman Coulter) for 5 minutes at room temperature in the dark. Unstained control samples were prepared in parallel to set gating parameters for live/dead discrimination.

Directly before analysis, samples were filtered through a 35 µm cell strainer to remove cell clumps. Flow cytometry was performed using Cytoflex Flow Cytometer (Beckman Coulter) and data were analyzed with the CytExpert software (v2.5, Beckman Coulter). At least 10,000 events were acquired per sample. Viable cells were defined as DAPI-negative, while non-viable cells were DAPI-positive.

**Immunofluorescence staining & imaging for 53BP1**

HepG2-NTCP cells (75,000/well) were plated on collagen-coated coverslips in 24-well plates. After 24 hours, cells were irradiated at 0.4 Gy X-ray and fixed 2 hours post-treatment with 4% PFA (158127, Sigma Aldrich) for 20 min at room temperature. Following 3 times of DPBS wash, cells were permeabilized with 0.25% Triton X-100 (Sigma Aldrich) for 30 min, then blocked and incubated with anti-53BP1 (1:100, ab21083, Abcam) in 1% BSA (A7906, Sigma Aldrich)/0.3% Triton X-100/DPBS at 4°C overnight. After DBPS wash, cells were stained with Alexa Fluor 594-conjugated secondary antibody (1:1000, A-11010, Abcam) and Hoechst-33342 (1:1000, 62249, ThermoFisher) in dark for 30 min, then mounted with ProLong Gold Antifade (P10144, Invitrogen). Imaging was performed on an Axio microscope (ZEISS Axio Imager 2, 68x oil objective) using fluorescence DAPI (blue)/TRITC(red) filters, with Z-stacking and optimized exposure to minimize background and capture multiple focal planes. The images were processed using ZEISS ZEN lite (Free Version).

**Results**

**Growth of cells exposed to X-ray** **irradiations to find appropriate dose**

To determine an X-ray dose that induces DNA damage without significantly impairing cell proliferation, we assessed the growth of HepG2-NTCP cells following exposure to increasing doses of X-ray irradiation (**Supplemental Figure 1A**). Cells were seeded in 24-well plates (Day 0), mock-infected with infection media containing 2.5% DMSO (Day 1), and irradiated with X-rays on Day 2 at doses of 0.08, 0.4, 2, or 4 Gy using the X-RAD320 Biological Irradiator (320 kVp, 12.5 mA, 2.0 mm Al filter, ~1.15 Gy/min). 2 hours post-irradiation, cells were trypsinised using TrypLE Enzyme and reseeded into 6-well plates for continued growth. On Day 5, cell viability and proliferation were assessed by flow cytometry.

To establish an optimal irradiation dose for inducing DNA damage while preserving cell viability, HepG2-NTCP cells were exposed to X-ray doses ranging from **0.08 to 4 Gy** and analyzed 72 hours post-treatment. Flow cytometry (CytoFlex, Beckman Coulter) revealed a **dose-dependent reduction in viability** (Supplemental Figure 1B). While **0.08 Gy** had negligible effects (viability comparable to non-irradiated controls), **0.4 Gy** caused a moderate reduction (93.16% vs. 93.57% live cells, Supplemental Figure 1D vs. 1C), striking a balance between DNA damage induction and retained proliferative capacity. In contrast, higher doses (**4 Gy**) significantly compromised viability (89.22% at 4 Gy, Supplemental Figure 1E), confirming cytotoxicity at therapeutic radiation levels.

The **0.4 Gy dose** was further validated by immunofluorescence staining of **53BP1 foci**, a marker of DNA double-strand breaks. X-ray irradiated HepG2-NTCP cells were fixed 2 hours post irradiation and exhibited discrete nuclear 53BP1 puncta (Supplemental Figure 1F), confirming DNA damage induction without catastrophic cell death. This aligns with the viability data and supports the use of **0.4 Gy** for subsequent integration experiments, where controlled DNA damage is required without overt toxicity.

**
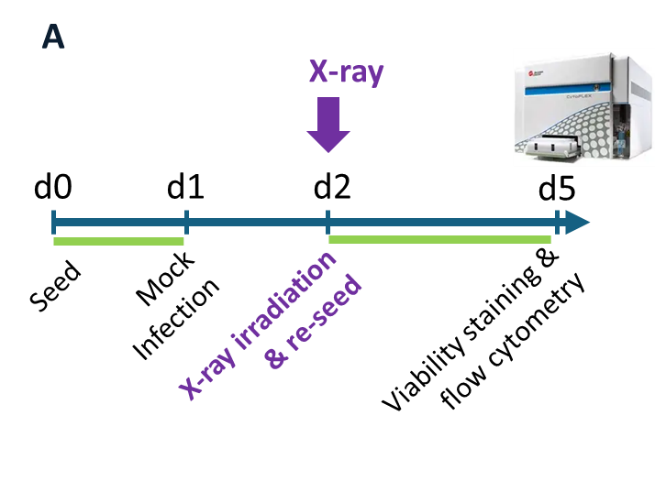

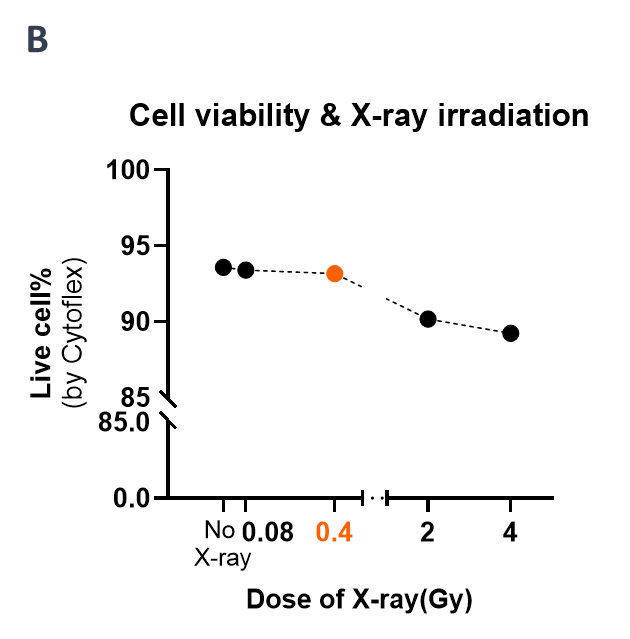
**


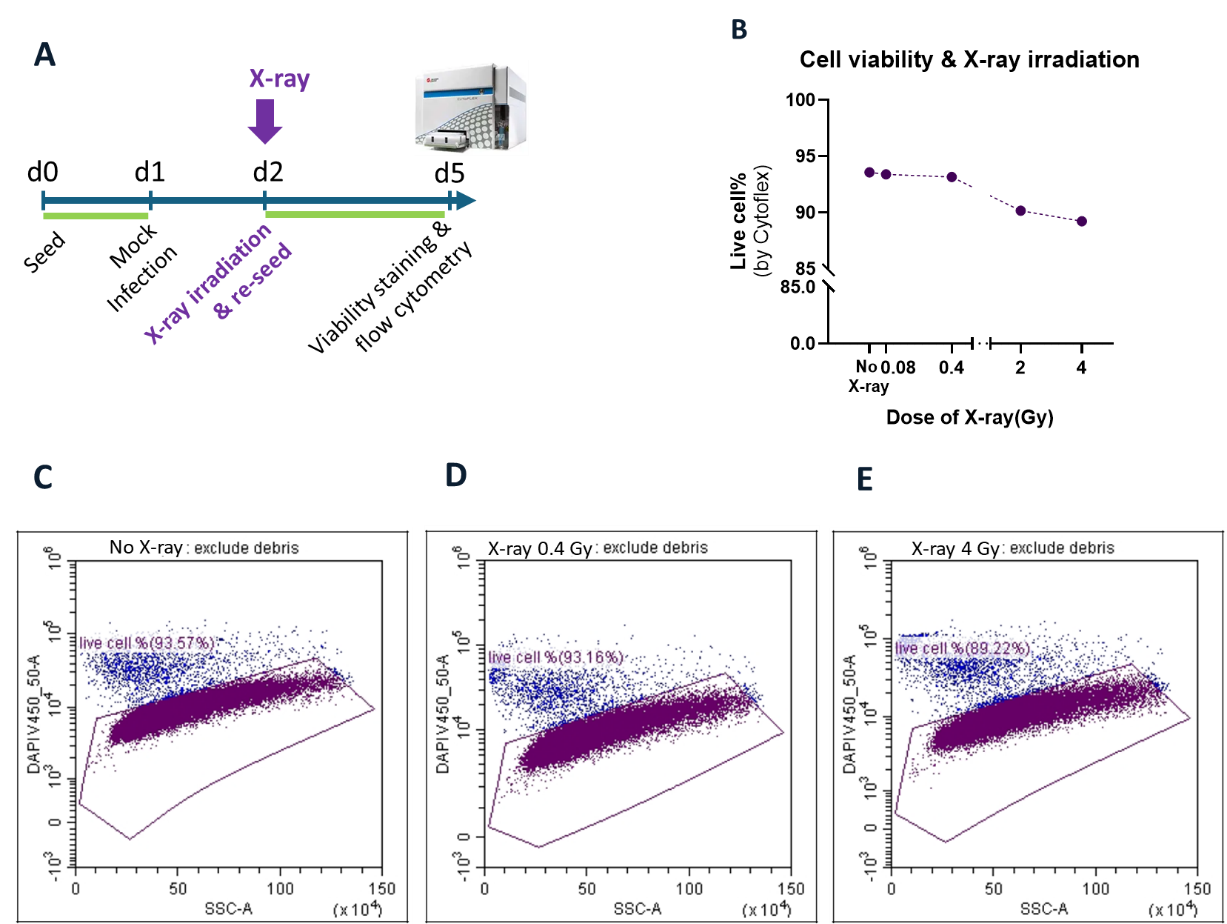


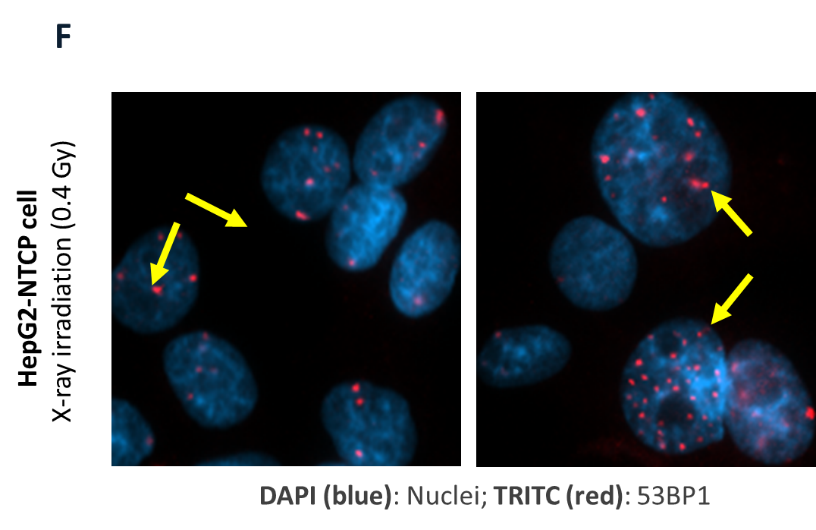


**Supplemental Figure 1. Determination of X-ray irradiation dose that is sufficient to induce DNA damage without significantly impairing cell viability or mitotic activity. (A)** **Experimental outline.** HepG2-NTCP cells were seeded and mock-infected, then irradiated with increasing doses of X-ray (0.08, 0.4, 2, or 4 Gy) on day 2 post-seeding. Two hours after X-ray irradiation, cells were re-seeded to support continued proliferation. Cell viability was assessed on day 5 using CytoFLEX flow cytometry. (**B)** **Cell viability by Cytoflex Flow Cytometer.** HepG2-NTCP cells were exposed to increasing doses of X-ray irradiation (0.08, 0.4, 2, and 4 Gy), and cell viability was assessed 3 days post-irradiation. The data show a dose-dependent reduction in viable cell populations. Cells exposed to 2 Gy and 4 Gy exhibited significantly decreased viability compared to the non-irradiated control, indicating cytotoxic effects at higher doses. In contrast, cells treated with 0.08 Gy showed viability comparable to control, while 0.4 Gy resulted in a moderate reduction, suggesting partial impact on proliferation without complete loss of viability. This supports the selection of 0.4 Gy (highlighted in orange) as an appropriate dose for inducing DNA damage while maintaining cell growth capacity. **(C-E) Representative flow cytometry dot plots (SSC-A vs. DAPI)** showing live cell populations under three irradiation conditions. C. Non-irradiated control: 93.57% live cells (DAPI-low, gate R1). D. 0.4 Gy irradiated: 93.16% live cells. E. 4 Gy irradiated: 89.22% live cells. Gates (R1) were set using unstained controls to define DAPI-low viable cells. Data were acquired on Cytoflex Flow Cytometer (Beckman Coulter) and data were analyzed with the CytExpert software (v2.5, Beckman Coulter). **(F) Example images showing DNA damage foci post-X-ray irradiation.** HepG2-NTCP cells irradiated with 0.4 Gy X-ray were fixed and immunostained for 53BP1 (DNA double-strand breaks marker). Merged fluorescence images (DAPI, blue; TRITC, red) show discrete nuclear 53BP1 foci (red puncta), suggestive of DNA double-strand breaks. Yellow arrows point to representative 53BP1 foci (red puncta). Images were acquired using a ZEISS Axio Imager microscope (63x oil objective) and processed with ZEISS ZEN lite. Scale bar: 10 µm.

**Cytotoxicity analysis for DNA inhibitors**

To determine the dose of compounds/DNA repair inhibitors that is sufficient to induce DNA damage without significantly impairing cell viability or mitotic activity. HepG2-NTCP cells were seeded and treated with ART558 (10, 20 or 40 nM), AZD6738 (0.5, 1 or 2 µM), NU7441(0.25, 0.5 or 1 µM) or VE822 (25, 50, 100, 200 or 400 nM) on day 0, then wash and mock-infected 24 hours post compound treatment (day 1), re-seeded on day 4 (3 days post mock-infection). Cell viability was assessed on day 7 using CytoFLEX flow cytometry (**Supplemental figure 2A**).

As shown in **Supplemental figure 2B-2E**, increasing concentrations of the selected inhibitors progressively reduced HepG2-NTCP cell viability, with higher doses causing marked cytotoxicity. To identify doses that induce cellular stress or DNA damage without significantly impairing cell viability or mitotic activity, we evaluated viability 5–7 days post-treatment using CytoFLEX flow cytometry. Based on these results, the following concentrations were selected for subsequent experiments: ART558 (10 nM), AZD6738 (0.5 µM), NU7441 (0.25 µM), and VE822 (50 nM). At these doses, cells maintained viability comparable to untreated controls, providing suitable conditions for downstream experiments assessing the effects of DNA repair pathway inhibition on HBV DNA integration.

**
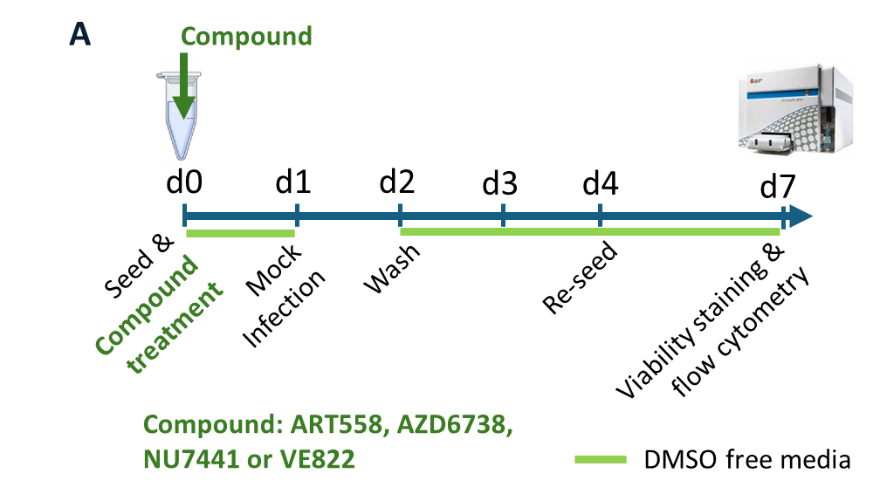
**

**
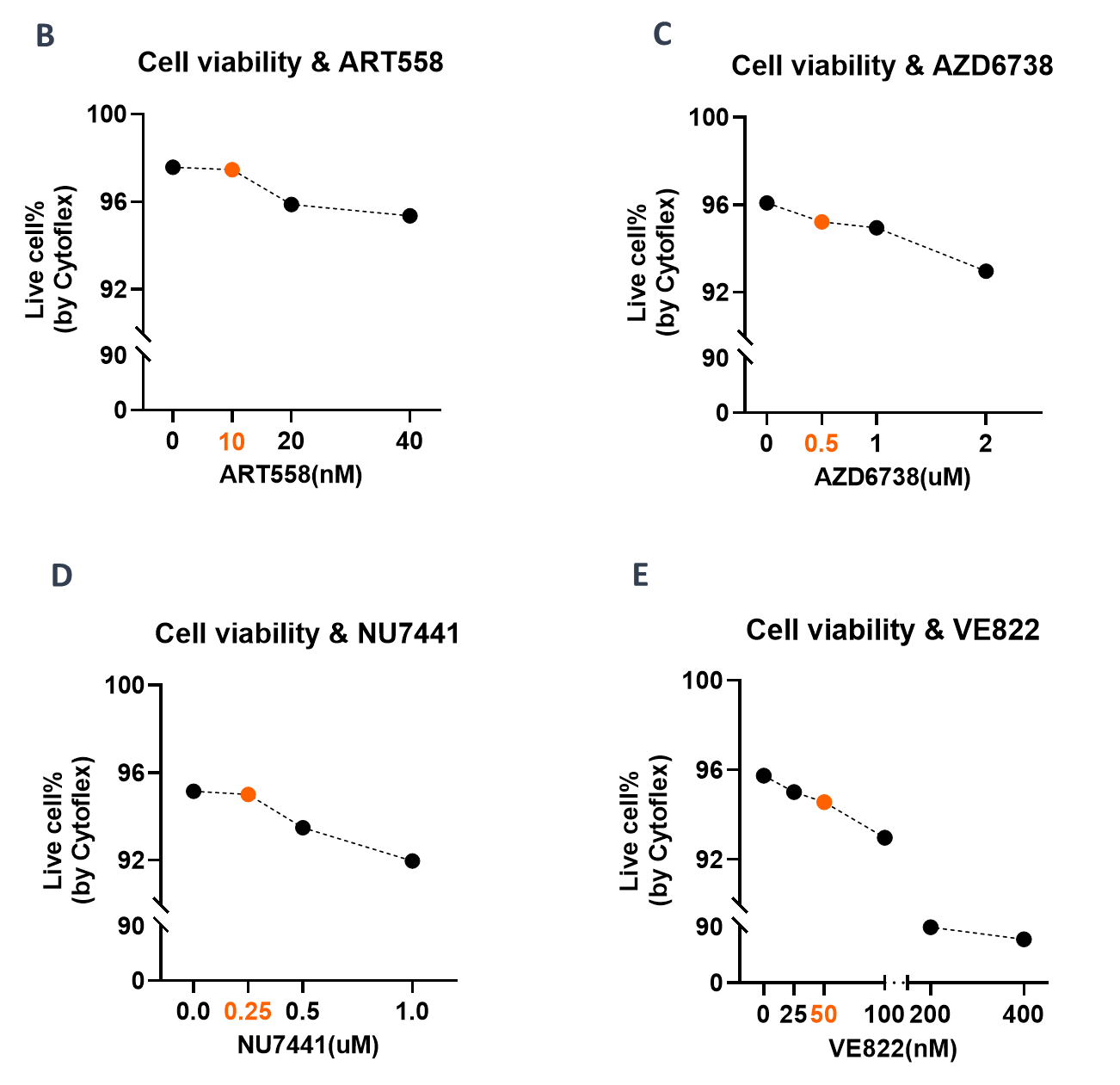
**

**
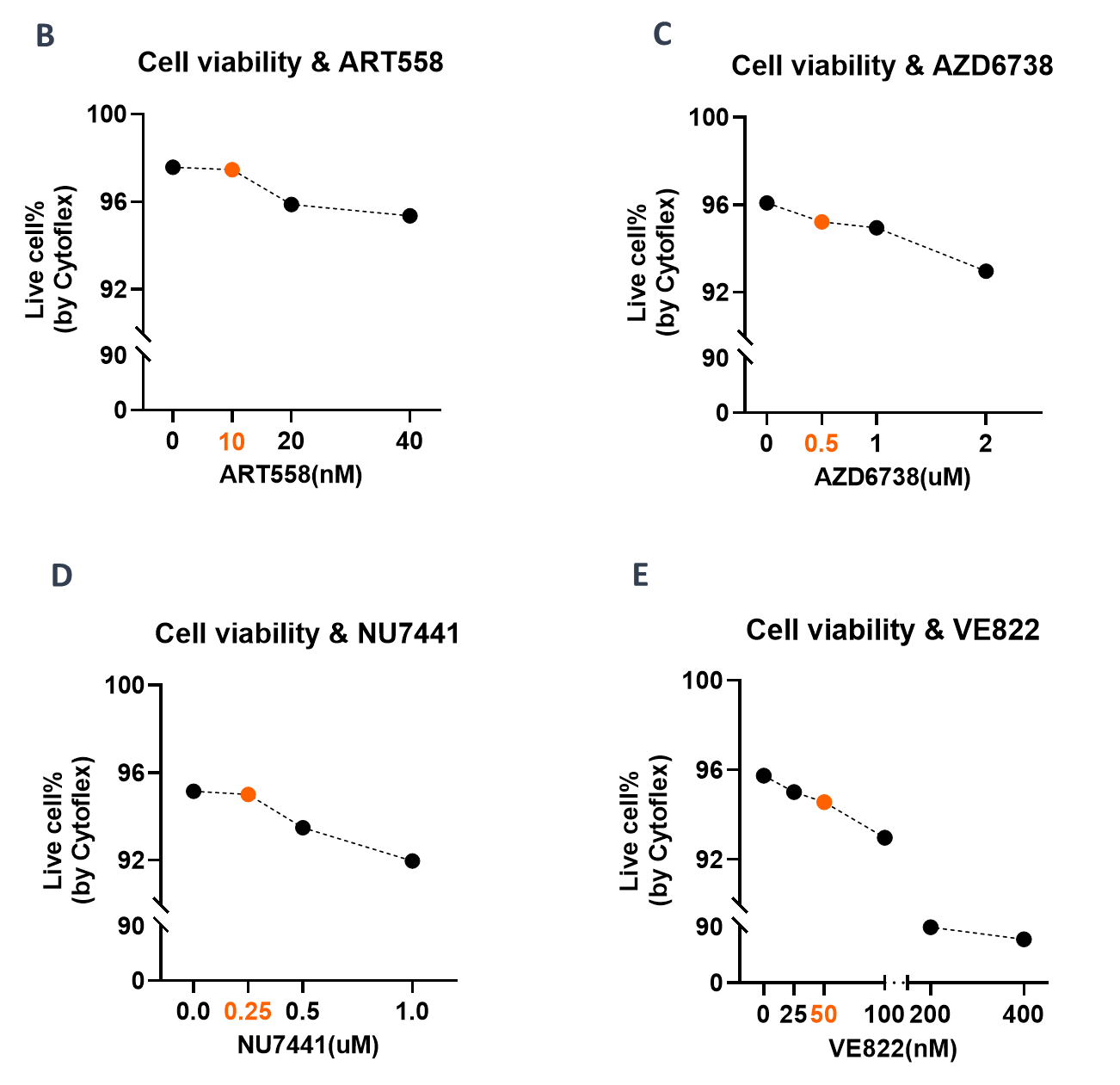
**

**Supplemental Figure 2.** **Determination of tolerable concentrations of DNA repair inhibitors in HepG2-NTCP cells.** **(A) Experimental outline.** HepG2-NTCP cells were seeded and treated with compounds on day 0, then mock-infected on day 1, then re-seeded on day 4 (3 days post mock-infection). Cell viability was assessed on day 7 using CytoFLEX flow cytometry. **(B-E) Cell viability by CytoFLEX flow cytometry (5-7 days post compounds treatment)**, treatment with increasing concentrations of DNA repair inhibitors resulted in dose-dependent reductions in HepG2-NTCP cell viability. High doses of VE822 (≥200 nM) and other compounds showed substantial cytotoxicity, while lower concentrations- ART558 (10 nM), AZD6738 (0.5 µM), NU7441 (0.25 µM), and VE822 (50 nM) had minimal effects on cell viability. These selected doses (highlighted in orange) were used for subsequent experiments, as they are well tolerated and allow investigation of DNA repair pathway modulation without confounding effects from excessive cytotoxicity.
